# Construction of Optimal Regeneration System for Chrysanthemum ‘11-C-2’ Stem Segment with Buds

**DOI:** 10.3390/plants13172403

**Published:** 2024-08-28

**Authors:** Qingbing Chen, Kang Gao, Bo Pan, Yaoyao Wang, Lijie Chen, Junjun Yu, Lili Wang, Yongming Fan, Haiying Li, Conglin Huang

**Affiliations:** 1 College of Architecture, North China University of Water Resources and Electric Power, Zhengzhou 450046, China; chenqb1228@163.com (Q.C.); panbo515@163.com (B.P.); clj_992@163.com (L.C.); fym126@126.com (Y.F.); lhy5100730@126.com (H.L.); 2 Institute of Grassland, Flowers and Ecology, Beijing Academy of Agriculture and Forestry Sciences, Beijing 100097, China; 16626702765@163.com (Y.W.); liliwang899@163.com (L.W.); 3 China United Engineering Corporation Limited, Hangzhou 310051, China; yujunjun@chinacuc.com

**Keywords:** tea chrysanthemum, tissue culture, induction proliferation, explant, regeneration system

## Abstract

*Chrysanthemum morifolium* ‘11-C-2’ is a variety of chrysanthemums with high ornamental and tea value, experiencing significant market demand. However, as cultivation areas expand, issues such as viral infection, germplasm degradation, low proliferation coefficient, and slow proliferation rate arise, necessitating the establishment of an efficient in vitro regeneration system. This study, based on the principles of orthogonal experimental design, explored the regeneration system of Chrysanthemum cultivar ‘11-C-2’ using sterile seedlings. The research focused on three key stages: adventitious bud differentiation, rooting culture, and acclimatization–transplantation, employing shoot-bearing stem segments and leaves as explants. The findings indicate that the optimal explant for the Chrysanthemum ‘11-C-2’ sterile seedlings is the shoot-bearing stem segment. The best medium for adventitious bud differentiation was determined to be MS supplemented with 1.5 mg/L 6-BA and 0.5 mg/L NAA. Bud differentiation began on day 17 with a 100% differentiation rate, completing around day 48. The maximum differentiation coefficient reached 87, with an average of 26.67. The adventitious buds were then cultured for rooting in the optimal medium of 1/2 MS supplemented with 0.1 mg/L NAA. Rooting was initiated on day 4 and was completed by day 14, achieving a rooting rate of 97.62%. After a 5-day acclimatization under natural light, the rooted seedlings were transplanted into a growth substrate with a peat-to-vermiculite ratio of 1:2. The plants exhibited optimal growth, with a transplantation survival rate of 100%. The findings provide data support for the efficient large-scale propagation of ‘11-C-2’ and lay the foundation for germplasm preservation and genetic transformation research of tea chrysanthemums.

## 1. Introduction

The chrysanthemum (*Chrysanthemum* × *morifolium* Ramat.), a perennial herbaceous plant of the Asteraceae family, boasts a documented history of over 4000 years in Chinese literature and more than 3000 years of cultivation [1,2]. With over 3000 known varieties [3], it not only holds significant ornamental value but also serves various functional purposes including tea-making, medicinal, and culinary applications [4,5,6]. Chrysanthemum ‘11-C-2’, a cultivar independently bred, possesses remarkable tea-making and ornamental qualities. Its yellow-white flowers naturally bloom in early September, exhibiting strong upright growth and branching habits, robust resistance, and adaptability, making it suitable for dense planting. Rich in flavonoids, volatile oils, amino acids, chlorogenic acid, and various micronutrients [7,8,9,10], it demonstrates extensive biological activities including antiviral, anti-inflammatory, anticancer, antioxidant, cardiovascular protective, anti-mutagenic, and anti-tumor effects [11,12,13,14,15,16,17,18], along with antimicrobial, analgesic, anti-fatigue, hypoglycemic, and hypolipidemic properties [19,20,21,22]. Currently, chrysanthemum propagation commonly relies on cutting and division methods, which are significantly influenced by environmental factors, resulting in low reproduction rates, slow propagation speed, and continuous viral accumulation [23,24,25]. This has led to functional degradation and reduced yields of chrysanthemum varieties [26,27,28,29], thereby impeding the industrial development of tea-use chrysanthemum [30,31,32]. Tissue culture technology offers a promising solution by enabling rapid propagation of high-quality plantlets in a short time and independent of environmental influences, significantly enhancing production efficiency [33]. Therefore, optimization of in vitro culture systems to ensure clean cultures and optimal media formulation and growth conditions is essential for further germplasm conservation and breeding efforts [34].

Many researchers have conducted extensive studies on the regeneration systems of Asteraceae plants, including *Chrysanthemum vestitum*, *Chrysanthemum* × *morifolium* ‘Jinbudiao’, *Senecio cruentus*, and *Chrysanthemum lavandulifolium*, as well as various tea and medicinal plants within the Asteraceae family such as *Taraxacum officinale* and *Saussurea involucrate* [35,36,37,38,39,40,41,42,43,44,45,46,47,48,49,50,51,52,53,54,55]. These regeneration systems typically add 1-Naphthylacetic acid (NAA) and N-(Phenylmethyl)-9H-purin-6-amine (6-BA) to the culture medium to quickly induce adventitious bud formation [35,36]. However, some Asteraceae plants, such as *Atractylodes lancea* and *Helenium aromaticum*, also require additional hormones like 2,4-Dichlorophenoxyacetic acid (2,4-D), kinetin (KT), and thidiazuron (TDZ) [55,56,57]. These regeneration systems typically use explants such as stem segments, leaves, hypocotyls, inter-nodal thin layers, and petals. The main factors affecting the regeneration of Asteraceae plants include the type of explant, the type and ratio of hormones in the culture medium, culture conditions, and the type of basic culture medium [58,59,60,61,62,63,64,65,66,67,68,69,70]. Additionally, genetic background diversity leads to significant differences in callus induction time, induction efficiency, regeneration rate, and regeneration coefficient among different Asteraceae species [63,71]. Therefore, regeneration systems are not universal [57], and the optimal culture medium for each stage of the efficient regeneration system for ‘11-C-2’ tea chrysanthemums still needs to be investigated. This involves the use of *Phalaenopsis aphrodite* [72], *Dioscorea opposita* [73], *Nematanthus glabra* [74], *Chrysanthemum morifolium* ‘Qiantouju5’ [75], *Lonicera edulis* Turcz. [76], and other plants at the stages of explant sterilization, healing tissue induction, adventitious shoot differentiation, and rooting culture. Acclimatization and transplantation are critical methods for propagating endangered plants and high-quality seedlings, influenced by factors such as plant condition, acclimatization duration, and transplantation substrate [77]. However, there is currently no research utilizing orthogonal experiments to investigate the optimal acclimatization duration and substrate composition.

This study employs orthogonal experimental design principles using bud-carrying stem segments and leaves of tea chrysanthemum ‘11-C-2’ as explants. It investigates the effects of different ratios of four plant growth hormones—6-BA, NAA, 2,4-D, and TDZ—on the proliferation and regeneration of ‘11-C-2’. The study identifies the optimal rooting medium, explores the best acclimatization period and transplantation substrate for ‘11-C-2’, and provides a theoretical basis for its large-scale rapid propagation and germplasm preservation. It also lays the groundwork for genetic transformation and molecular mechanism research related to tea chrysanthemums.

## 2. Results

### 2.1. Optimal Medium for Adventitious Bud Differentiation of ‘11-C-2’

According to the R and k values of the differentiation coefficient in Appendix A. Table A1, the primary factors influencing the differentiation coefficient of bud-carrying stem segments of ‘11-C-2’ are 6-BA concentration > 2,4-D concentration > NAA concentration > TDZ concentration. The optimal levels for these factors are ranked as A2 > A3 > A1, B3 > B1 > B2, C1 > C2 > C3, D1 > D2 > D3. The effects of different levels of these factors on the differentiation coefficient of ‘11-C-2’ shoot segments are highly significant (*p* < 0.001). The optimal combination of factors in the experiments for influencing the differentiation coefficient is A2B3C1D2 Table 1, which corresponds to MS + 1.5 mg/L 6-BA + 0.5 mg/L NAA + 0.1 mg/L TDZ. However, the range analysis shows the optimal combination as A2B3C1D1, which corresponds to MS + 1.5 mg/L 6-BA + 0.5 mg/L NAA.

For the leaves of ‘11-C-2’, the primary factors influencing the differentiation coefficient are NAA concentration > 6-BA concentration > TDZ concentration > 2,4-D concentration. The optimal levels for these factors are ranked as A2 > A1 > A3, B1 > B2 > B3, C1 > C2 > C3, D1 = D2 > D3. However, the effects of these factors at different levels on the differentiation coefficient of ‘11-C-2’ leaves are not significant (*p* = 0.454). The optimal combination of factors in the experiments for influencing the differentiation coefficient is A1B1C1D1 Table 1, which corresponds to MS + 1.0 mg/L 6-BA + 0.1 mg/L NAA. However, the range analysis shows the optimal combination as A2B1C1D1 or A2B1C1D2. To reduce hormone usage, TDZ is set at the first level of 0 mg/L, corresponding to MS + 1.5 mg/L 6-BA + 0.1 mg/L NAA. The optimal combinations for both bud-carrying stem segments and leaves are not included in the nine treatments listed in Table 1.

According to Table 2, the concentrations of 6-BA, NAA, 2,4-D, and TDZ have a highly significant impact on the differentiation coefficient of bud-carrying stem segments of ‘11-C-2’ (*p* < 0.001). The analysis of variance results is consistent with the range analysis results, indicating that the optimal medium for adventitious bud differentiation for ‘11-C-2’ bud-carrying stem segments is MS + 1.5 mg/L 6-BA + 0.5 mg/L NAA. However, the concentrations of these four hormones do not show significant differences in their impact on the differentiation coefficient of ‘11-C-2’ leaves (*p* > 0.05), making it impossible to determine the optimal hormone ratio for the culture medium.

**Figure 1:**
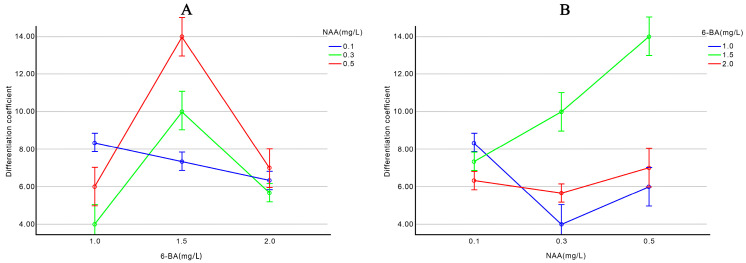
Effect of 6-BA (**A**) and NAA (**B**) concentrations on the differentiation coefficient of stem segments with buds.

According to Appendix A. Table A2, there is a highly significant interaction effect between the concentrations of 6-BA and NAA on the differentiation coefficient of bud-carrying stem segments. When the NAA concentration is 0.1 mg/L, the differentiation coefficient decreases as the 6-BA concentration increases. However, when the NAA concentration is 0.3 mg/L or 0.5 mg/L, the differentiation coefficient first increases and then decreases with increasing 6-BA concentration, peaking at 1.5 mg/L 6-BA Figure 1A. When the 6-BA concentration is 1.0 mg/L or 1.5 mg/L, the differentiation coefficient first decreases and then increases with increasing NAA concentration. At 1.5 mg/L 6-BA, the differentiation coefficient increases with increasing NAA concentration, peaking at 0.5 mg/L NAA Figure 1B. However, low concentrations of NAA or high concentrations of 6-BA are not suitable for adventitious bud differentiation in bud-carrying stem segments.

**Figure 2:**
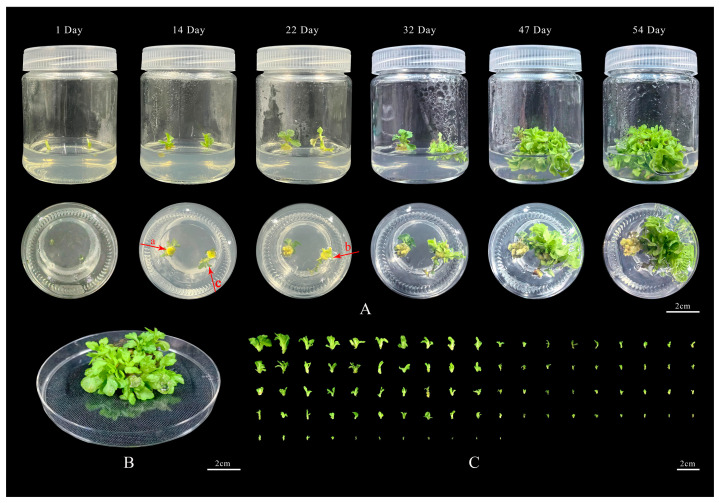
Illustrates the adventitious bud differentiation of ‘11-C-2’ stem segments. (**A**) Callus induction and adventitious bud differentiation process using stem segments as explants (Bar = 2 cm); (**B**) Completed state of adventitious bud differentiation (Bar = 2 cm); (**C**) Maximum differentiation coefficient of ‘11-C-2’ stem segments (Bar = 2 cm); (a) Formation of callus at the cut end of the budded stem segment; (b) The callus starts to differentiate into adventitious buds; (c) Axillary buds on the stem segment directly differentiate into adventitious buds.

In summary, the optimal explant for ‘11-C-2’ is the budded stem segment. Validation of the optimal combination identified in the analysis revealed that when this explant is cultured on the optimal medium for adventitious bud differentiation, which is MS + 1.5 mg/L 6-BA + 0.5 mg/L NAA (combination A2B3C1D1). Callus formation occurs at the cut ends of the stem segments within 9 days. The callus then differentiates into adventitious buds within 17 days. Additionally, axillary buds on the stem segments can directly develop into adventitious buds within 12 days, with a differentiation rate of 100% Figure 2A. Complete differentiation of adventitious buds is achieved within 48 days Figure 2B, with an average differentiation coefficient of 26.67 and a maximum of 87 Figure 2C, significantly higher than combination A2B3C1D2, and the buds show vigorous growth.

### 2.2. Optimal Rooting Medium for ‘11-C-2’

Based on Table 3 and Appendix A. Table A1, the main factors affecting the number of roots and root length in ‘11-C-2’ plantlets are the NAA concentration and the basal medium, with NAA concentration being more significant. The optimal levels for these factors are in the order of A2 > A3 > A1 and B1 > B2 > B3. The effect of different levels of these factors on root number and length is highly significant (*p* < 0.001). The primary factors affecting the rooting rate of ‘11-C-2’ plantlets are also the NAA concentration and the basal medium, with NAA concentration being more influential. However, the optimal levels for rooting rate are in the order of A3 > A2 > A1 and B1 > B2 > B3. The variation in these factors’ levels does not significantly impact the rooting rate (*p* = 0.943). Therefore, the optimal combination for promoting root development in ‘11-C-2’ plantlets is A2B1, which corresponds to 1/2 MS medium supplemented with 0.1 mg/L NAA. This optimal combination is included in the 9 treatments listed in Table 3.

According to Table 4, the base medium and NAA concentration have a highly significant effect on the root number and root length of ‘11-C-2’ (*p* < 0.001), but no significant effect on the rooting rate (*p* > 0.05). The results of the variance analysis are consistent with the range analysis results, indicating that the optimal rooting medium for ‘11-C-2’ is 1/2 MS + 0.1 mg/L NAA.

**Figure 3:**
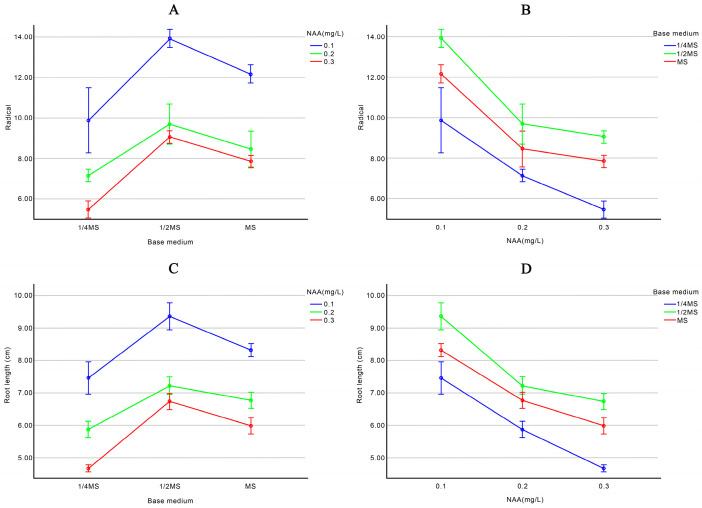
Effects of basal medium and NAA concentration on root length and root number. (**A**) The effect of basic medium on the number of seedlings; (**B**) Effect of NAA concentration on the number of seedling roots; (**C**) Effect of base medium on root length of seedling; (**D**) Effect of NAA concentration on root length of seedling.

According to Appendix A. Table A2, there is a highly significant interaction effect between the base medium and NAA concentration on the changes in root number and root length of rooting seedlings. When the NAA concentration is constant, varying the base medium results in a trend where root number and length first increase and then decrease, with the highest root number and length observed with 1/2 MS as the base medium. Conversely, when the base medium is constant, increasing the NAA concentration leads to a decreasing trend in both root number and length, with the maximum root number and length observed at an NAA concentration of 0.1 mg/L Figure 3.

**Figure 4:**
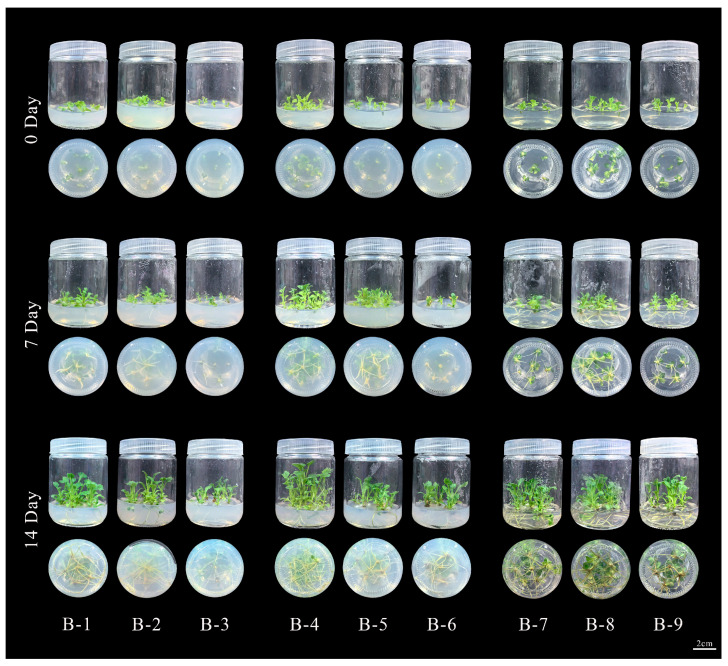
Illustrates the rooting status of ‘11-C-2’ adventitious shoots under various treatments. Rooting and plant status of ‘11-C-2’ adventitious buds at 0–14 days under different treatments, Bars = 2 cm.

**Figure 5:**
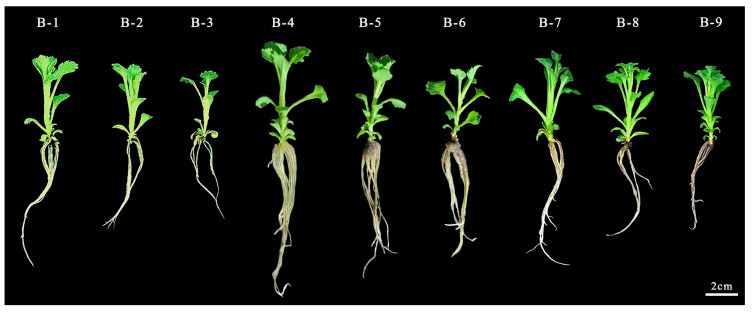
Morphological appearance of tissue-cultured plantlets after 14 days of rooting under different treatments. Bar = 2 cm.

In summary, the optimal rooting medium for ‘11-C-2’ in the experimental treatments is 1/2 MS + 0.1 mg/L NAA. The adventitious buds of ‘11-C-2’ are capable of normal rooting across all nine media tested in Figure 4. Comparative analysis of rooting morphology for representative seedlings from each treatment Figure 5 shows that the optimal treatment has superior root length, root number, and plant condition compared to others. This indicates that a small amount of NAA added to a suitable base medium can promote rooting in ‘11-C-2’, resulting in healthy seedlings, whereas excessive NAA inhibits rooting and slows seedling growth.

### 2.3. Optimal Hardening Time and Transplant Medium for ‘11-C-2’

According to Appendix A. Table A1, the main factors affecting the height of ‘11-C-2’ plants are hardening time > transplant medium (0–10 d) and transplant medium > hardening time (10–15 d). The optimal levels for these factors are ranked as A3 > A2 > A1 (0–15 d), B3 > B2 > B1 (0 d), and B2 > B3 > B1 (5–15 d). However, the differences in plant height at 0 days are not significant (*p* = 0.198). Therefore, the optimal combination for influencing the height of ‘11-C-2’ plants in the experimental treatments is A3B2, meaning transplanting into a medium with a 1:2 ratio of peat to perlite after 5 days of hardening results in the best growth. This optimal combination is included in the nine treatments listed in Table 5.

According to Table 6, there are no significant differences in plant height (0 days) due to hardening time and transplant medium. However, these factors have highly significant differences in plant height (5 days) (*p* < 0.001) and very significant differences (*p* < 0.01) in plant height (10 days) and (15 days) (*p* < 0.001). The results of the variance analysis are consistent with those of the range analysis, indicating that the optimal hardening time for ‘11-C-2’ is 5 days and the optimal transplant medium is a 1:2 ratio of peat to perlite.

**Figure 6:**
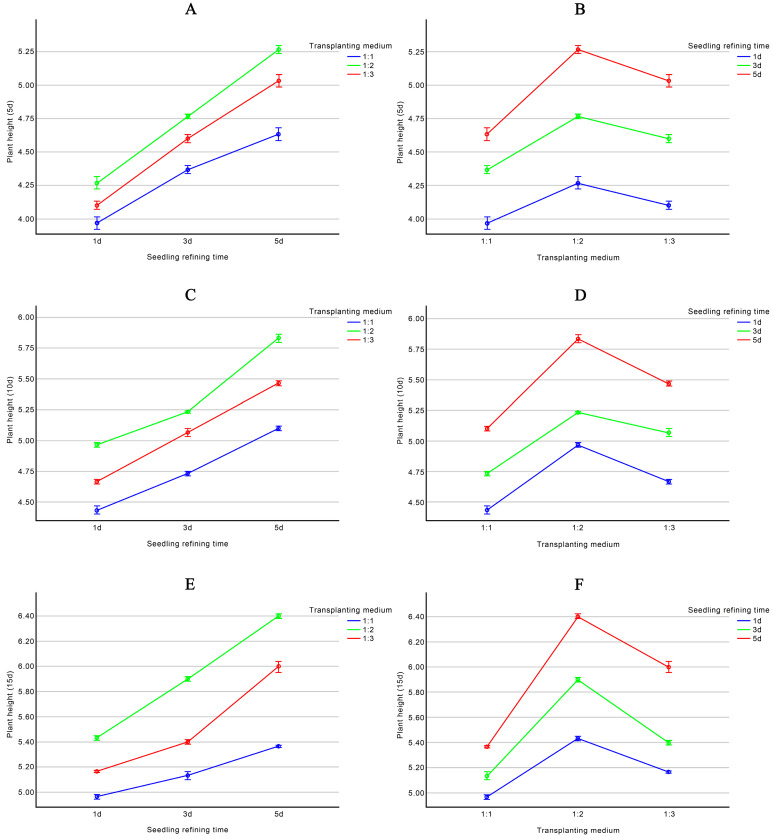
Effects of seedling refining time and transplanting medium on plant height. (**A**) Effect of seedling refining time on plant height (5 d); (**B**) Effect of transplanting medium on plant height (5 d); (**C**) Effect of seedling refining time on plant height (10 d); (**D**) Effect of transplanting medium on plant height (10 d); (**E**) Effect of seedling refining time on plant height (15 d); (**F**) Effect of transplanting medium on plant height (15 d).

According to Appendix A. Table A2, there is a highly significant interaction effect between hardening time and transplant medium on plant height changes. When the transplant medium ratio is constant, the height of ‘11-C-2’ increases continuously from 0 to 15 days, with the tallest plants observed after 5 days of hardening. Conversely, when the hardening time is constant, varying the transplant medium ratio results in a trend where plant height first increases and then decreases from 0 to 15 days, with the tallest plants observed at a peat-to-perlite ratio of 1:2 in Figure 6.

**Figure 7:**
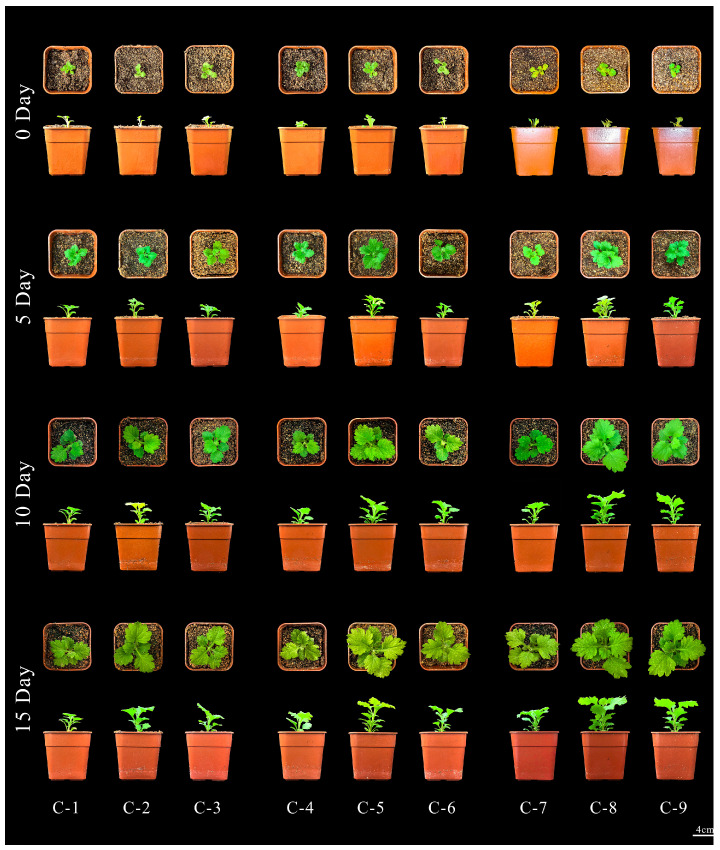
Illustrates the growth status of ‘11-C-2’ under different substrate ratios. ‘11-C-2’ growth of root-rooted seedlings after transplanting 0–15 d; Bar = 4 cm.

In summary, the optimal hardening time for ‘11-C-2’ in the experimental treatments is 5 days, and the best transplant medium is a 1:2 ratio of peat to perlite. Rooted ‘11-C-2’ seedlings gradually acclimate to the external environment by slowly increasing the opening of the culture bottles under natural light. Both hardening and transplant survival rates can reach 100%, with the transplantation process completed in about 15 days Figure 7. During the early transplant period (0–10 d), longer hardening times enhance ‘11-C-2’ growth, while in the later period (10–15 d), the transplant medium ratio primarily affects the growth of ‘11-C-2’.

### 2.4. Establishment of ‘11-C-2’ Regeneration System with Sprouted Stem Segments

**Figure 8:**
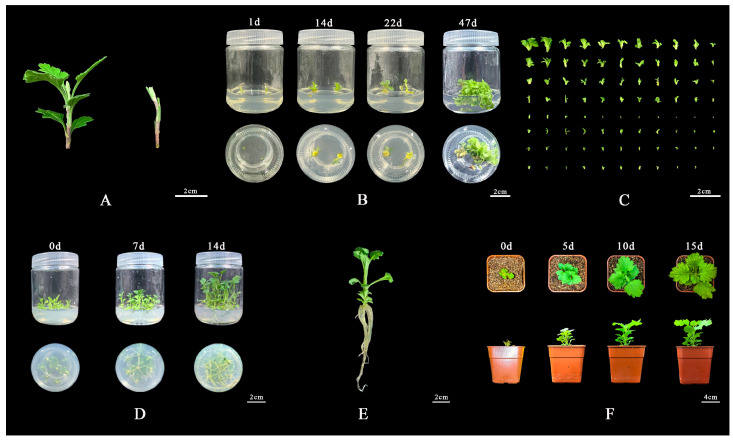
‘11-C-2’ Regeneration process of sprouted stem segments. (**A**) Explant, Bar = 2 cm; (**B**) Callus induction and adventitious bud differentiation, Bar = 2 cm; (**C**) Adventitious bud differentiation is complete, Bar = 2 cm; (**D**) Rooting culture, Bar = 2 cm; (**E**) Root plant, Bar = 2 cm; (**F**) Hardening and transplanting, Bar = 4 cm.

Based on the results from the stages of shoot regeneration, rooting, and acclimatization, this study has developed an initial regeneration system for tea chrysanthemum ‘11-C-2’ using shoot segments with buds as explants Figure 8. The shoot segments were inoculated onto the optimal medium for adventitious bud differentiation, MS + 1.5 mg/L 6-BA + 0.5 mg/L NAA (Figure 8A). Over 5 days, the shoot segments gradually changed from light green to green. Approximately 2 days later, the basal cut surface of the shoot segments began to swell, and after 1–3 days, a yellow–green callus formed at the base of the swollen segments. After continuing the culture for about 8 days, the callus started to differentiate into adventitious buds. Buds from axillary shoots differentiated directly after 12 days, completing the differentiation process in approximately 48 days Figure 8B, with an average differentiation coefficient of 26.67 and a maximum of 87 Figure 8C. Once the adventitious shoots had reached a length of 2 cm, ‘11-C-2’ adventitious shoots were excised and inoculated in the optimal rooting medium of 1/2 MS + 0.1 mg/L NAA. A concentration of 1 mg/L NAA was employed, and the adventitious shoots began to root successively within four days. The root system was stout and dense in general, with most of the roots exhibiting a yellowish hue and a few displaying a white coloration. The rooting rate was 97.62%, and the rooting culture could be completed within 14 days Figure 8D. The rooted seedlings exhibited a good growth condition in general Figure 8E. Subsequently, the rooted seedlings were gradually opened under natural light for a period of five days. Following this, they were transplanted into the growth substrate, with a mass ratio of charcoal:vermiculite of 1:2. The plants exhibited robust growth, and the survival rate of the transplanting reached 100% in Figure 8F.

## 3. Discussion

Plant tissue culture is a crucial technique for conserving endangered species and maintaining plant quality, but it is influenced by various factors such as explant type, medium composition, and plant hormone formulations. The choice of explant is fundamental in plant regeneration. Different explants from the same plant, depending on their growth stage and physiological characteristics, exhibit variations in callus induction, regeneration rate, and regeneration coefficient [60,78,79]. For chrysanthemums, explants such as petals, petioles, axillary buds, hypocotyls, and shoot tips can be used [80,81,82,83,84,85]. However, shoot segments and leaves are widely used due to their ease of collection and abundance [86,87]. In this study, we found that the basal cut of shoot segments with buds form calluses and differentiate into adventitious buds, while axillary buds on the shoot segments directly differentiate into adventitious buds, resulting in a shorter differentiation time and higher coefficient. In contrast, leaf cuttings take longer to form callus and differentiate into adventitious buds, with a lower differentiation coefficient, consistent with previous studies [88,89].

In tissue culture, the medium contains specific ratios of cytokinins and auxins. High concentrations of cytokinins promote adventitious bud formation, high concentrations of auxins favor root formation, and a specific balance of both hormones is optimal for callus formation [79,90]. For chrysanthemum species, MS medium is commonly used for adventitious bud differentiation [91], often supplemented with hormones like 6-BA, NAA, KT, or TDZ to determine the best hormone combination by adjusting the ratio and types of auxins and cytokinins [88,92,93]. Established regeneration systems for chrysanthemums typically use 6-BA and NAA to induce adventitious buds [36,94,95,96]. This study found that high concentrations of 6-BA and low concentrations of NAA are not suitable for callus induction and bud differentiation in ‘11-C-2’, consistent with findings that high 6-BA concentrations inhibit bud differentiation in other species like chrysanthemum and Helenium aromaticum [57,93]. Additionally, 2,4-D and TDZ are unsuitable for ‘11-C-2’ callus induction and bud differentiation, contrary to results reported by Luo Hong [57]. The differentiation times, bud differentiation rates, and differentiation coefficients also vary among different chrysanthemum varieties [34,97]. In this study, bud-bearing stem segments of “11-C-2” can differentiate into adventitious buds within 17 days, with a differentiation rate of 100% and a coefficient of 26.7. However, leaf segments take longer to differentiate into adventitious buds, with a differentiation coefficient of only 2. This indicates that, compared to leaf segments, stem segments in some chrysanthemum varieties have a shorter differentiation time and a higher coefficient, making them more suitable as explants.

In chrysanthemum root culture, a common approach is to use 1/2 MS basic medium with low concentrations of NAA to stimulate root growth [87,98,99]. However, either omitting NAA or using high concentrations of NAA can also promote root formation in chrysanthemums [57,81]. Other hormones like IBA and IAA are also used and can achieve good rooting results [100,101]. This study found that a small amount of NAA promotes rooting in ‘11-C-2’ adventitious buds, while either omitting NAA or using excessively high concentrations negatively affects root length, number, and plant growth. This finding is consistent with previous research and suggests that while the optimal rooting medium varies among most chrysanthemum species, some share similar rooting media.

After removing chrysanthemum-rooted seedlings from culture bottles, changes in the growth environment can significantly impact their survival rate. Therefore, acclimation is necessary before transplanting. Studies have shown that seedlings have the highest survival rate after 3 days of acclimation [102], with survival rates decreasing if the duration is shorter or longer [103]. In this study, ‘11-C-2’ seedlings achieved a 100% survival rate after 5 days of acclimation, indicating strong adaptability and stress resistance. Suitable acclimation substrates include vermiculite, perlite, and decomposed straw [104], or a peat and coconut coir mix in equal parts [105]. By adjusting the ratio of vermiculite to peat, this study found that a 1:2 ratio of peat to vermiculite provided the best growth conditions for the seedlings, with a 100% survival rate. This suggests that seedlings can adapt more quickly to external conditions when grown on a substrate with appropriate aeration and water retention.

In summary, this study demonstrates that shoot tip segments of ‘11-C-2’ are the most suitable explants for adventitious bud differentiation. Using appropriate concentrations of 6-BA and NAA, successful differentiation is achieved. The effects of these hormones on bud differentiation have been clearly defined. Additionally, the study identified the optimal rooting medium from various combinations of basic media and NAA concentrations, enhancing our understanding of the role of media, hormone types, and concentrations in plant tissue culture. Based on orthogonal experiments, the optimal acclimatization duration and transplantation substrate were efficiently determined, adding valuable insights into the acclimatization and transplantation stages for chrysanthemum species. The developed high-efficiency propagation system for ‘11-C-2’ using shoot tip segments integrates results from all three stages. Future work should focus on optimizing this system to improve its stability and efficiency, laying the groundwork for developing genetic transformation systems and gene function validation using ‘11-C-2’. Furthermore, plant tissue culture can remove certain chrysanthemum viruses, and this study provides a foundation for establishing efficient virus-free seedling propagation techniques for tea chrysanthemum.

## 4. Materials and Methods

### 4.1. Experimental Material

**Figure 9:**
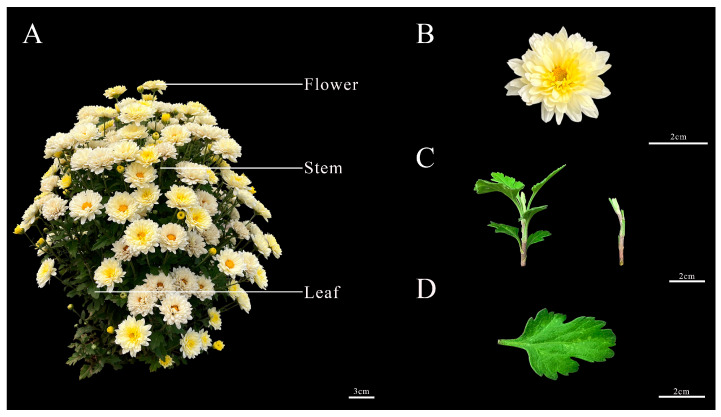
Morphology of the whole plant and floral organs of Chrysanthemum ‘11-C-2’. (**A**) Whole plant (Bar = 3 cm); (**B**) Capitulum (Bar = 2 cm); (**C**) Stem segment with leaf and stem segment (Bar = 2 cm); (**D**) Leaf blade (Bar = 2 cm).

The Chrysanthemum ‘11-C-2’ material used in this experiment was autonomously bred by the Laboratory of Forage and Ornamental Horticulture, Beijing Academy of Agriculture and Forestry Sciences as shown in Figure 9.

### 4.2. Experimental Method

#### 4.2.1. Aseptic Seedling Culture

Select healthy ‘11-C-2’ chrysanthemum plants in the vegetative growth stage and cut the stems into segments with two axillary buds each Figure 9C. Immerse the segments in a 3% detergent solution for 15 min, followed by shaking on a shaker for 10 min. Rinse the segments three times with sterile water, then quickly disinfect with 75% alcohol, followed by three more rinses with sterile water. Next, sterilize the segments in a 5% sodium hypochlorite solution for 15 min and rinse them three times with sterile water. Place the sterilized segments on sterile absorbent paper in a sterile Petri dish. In each 250 mL culture bottle, add 50 mL of MS medium without any hormones and inoculate with five segments. After 25 days of aseptic culture, a large number of ‘11-C-2’ chrysanthemum aseptic seedlings are obtained. Culture conditions: 25 °C temperature, 1500 lx light intensity, and photoperiod (16 h light/8 h dark).

#### 4.2.2. Adventitious Bud Differentiation

Using the sterile seedlings of tea chrysanthemum ‘11-C-2’ as experimental materials, the stems were cut into 1 cm segments containing buds, and the leaves were cut into 0.5 cm × 0.5 cm pieces as explants. After undergoing the previously described disinfection and sterilization process, the explants were inoculated onto MS basal medium supplemented with different concentrations of 6-BA, NAA, 2,4-D, and TDZ. Following the principles of orthogonal experimental design, nine different hormone combinations were formulated for adventitious bud differentiation in Appendix A. Table A5. For each experimental group, five 250 mL culture flasks were used, each containing 50 mL of the medium and inoculated with two explants, with three replicates, resulting in a total sample size of N = 30 per group. During the adventitious bud differentiation culture, the state of the explants was monitored. After 54 days of cultivation, the differentiation coefficient of the adventitious buds for ‘11-C-2’ was recorded, and the optimal hormone combination for the differentiation medium was identified. Since the orthogonal test represents all possible experimental combinations by selecting a subset of representative combinations, if the optimal hormone combination is not included in the initial test, further experiments are required to validate the selected optimal combination to ensure the accuracy of the experimental results. The cultivation conditions were the same as those described in Section 4.2.1.

#### 4.2.3. Rooting Culture

After 54 days of differentiation culture of ‘11-C-2’ adventitious buds Figure 2B, the resulting buds were grown to a height of 2 cm and had developed two leaves. These buds were then separated into individual units using sterile tweezers Figure 2C and transferred to rooting media containing varying concentrations of NAA on different basal media. In accordance with the orthogonal experimental design, nine different rooting media treatments were established in Appendix A. Table A6. Each treatment consisted of three 250 mL culture flasks, each containing 50 mL of medium and inoculated with six adventitious buds. The experiment was repeated three times, yielding a total sample size of N = 54 per group. During the rooting culture, the growth status of the plants and root development were observed. After 14 days of culture, the rooting rate, root length, and the number of roots of ‘11-C-2’ seedlings were recorded. The cultivation conditions were the same as described in Section 4.2.1.

#### 4.2.4. Acclimatization and Transplantation

Under natural light conditions, the caps of the ‘11-C-2’ rooting bottles are gradually opened, first slightly, then halfway, and finally completely, to acclimatize the plants for different durations. After acclimatization, seedlings are carefully removed from the bottles using sterile tweezers. The roots are washed with water to remove the residual medium, and seedlings measuring 3 cm in height with 5 leaves are treated with 50% Carbendazim solution for 2 min. Then, the seedlings are planted into growth soil with varying ratios of peat moss to vermiculite. According to orthogonal experimental design principles, 9 different treatments are set up see Table A7. Each treatment includes 10 planting pots (6 cm × 6 cm × 8 cm), with one plant per pot, and each treatment is repeated three times, resulting in a total of 30 samples per group. Watering is performed every 5 days with 150 mL, and plant growth is observed. Plant height is measured at 0, 5, 10, and 15 days, and survival rates are calculated at 15 days. Cultivation conditions: temperature 25 °C, humidity 50%, with gradually increasing ventilation during acclimatization.

### 4.3. Data Analysis

In this study, the data were organized using Microsoft Excel, and the experimental data were analyzed by ANOVA and simple effects analysis using the SPSS 27.0 software. The multiple comparisons were conducted using Duncan method, and the results were expressed as “mean ± standard deviation”. The following formulas were utilized for calculation:

Differentiation rate = (Number of explants producing adventitious buds/total number of uncontaminated explants in the same treatment) × 100%;

Differentiation coefficient = (Total number of adventitious buds differentiated/Total number of uncontaminated explants in the same treatment) × 100%;

Rooting rate = (Number of rooted plantlets/Total number of uncontaminated plantlets in the same treatment) × 100%;

Transplant survival rate = (Number of surviving transplants/Total number of transplants) × 100%.

## 5. Conclusions

Based on the results from the stages of adventitious bud differentiation, rooting, and acclimatization, this study successfully established a highly efficient propagation system for tea chrysanthemum ‘11-C-2’ using shoot tip segments as explants. The optimal differentiation medium is MS with 1.5 mg/L 6-BA and 0.5 mg/L NAA, while the best rooting medium is 1/2 MS with 0.1 mg/L NAA. The ideal acclimatization period is 5 days, and the most effective transplantation substrate is a peat ratio of 1:2. This system achieves bud differentiation in 48 days with a 100% differentiation rate and an average coefficient of 26.67, reaching up to 87. Rooting is completed in 14 days with a rooting rate of 97.62% and healthy root development. Acclimatization and transplantation are achieved in 20 days with a 100% survival rate and strong plant growth. This method minimizes environmental impacts, enables year-round production, significantly enhances the propagation efficiency of tea chrysanthemum ‘11-C-2’, and shortens the breeding cycle, providing a robust foundation and data support for its large-scale cultivation and application.

## Figures and Tables

**Table 1:** ‘11-C-2’ adventitious bud differentiation results.

Treatment	Factor A	Factor B	Factor C	Factor D	Differentiation Coefficient	
	6-BA (mg/L)	NAA (mg/L)	2,4-D (mg/L)	TDZ (mg/L)	Stem Segment with Bud	Leaf
A-1	1.0	0.1	0	0	8.33 ± 0.58 c	2.00 ± 1.00 a
A-2	1.0	0.3	0.3	0.1	4.00 ± 1.00 f	1.33 ± 0.58 a
A-3	1.0	0.5	0.1	0.3	6.00 ± 1.00 de	1.00 ± 0 a
A-4	1.5	0.1	0.3	0.3	7.33 ± 0.58 cd	1.67 ± 0.58 a
A-5	1.5	0.3	0.1	0	10.00 ± 1.00 b	1.67 ± 0.58 a
A-6	1.5	0.5	0	0.1	14.00 ± 1.00 a	1.67 ± 0.58 a
A-7	2.0	0.1	0.1	0.1	6.33 ± 0.58 de	1.67 ± 0.58 a
A-8	2.0	0.3	0	0.3	5.67 ± 0.58 e	1.00 ± 1.00 a
A-9	2.0	0.5	0.3	0	7.00 ± 1.00 cde	1.00 ± 0 a

The differentiation coefficients were compared by Duncan method, with results expressed as “mean ± standard deviation”. Different lowercase letters indicate highly significant differences (*p* < 0.001), but there was no significant difference among the variation at 5% of One-way ANOVA in leaf differentiation coefficient (*p* = 0.454). The total number of explants per experimental treatment was N = 30.

**Table 2:** ANOVA of ‘11-C-2’ differentiation coefficient.

Dependent Variable	Source of Variance	Sum of Squares	Mean Square	F	*p*
Differentiation coefficient (Stem segment with bud)	6-BA	107.185	53.593	76.158 ***	0.000
	NAA	28.074	14.037	19.947 ***	0.000
	2,4-D	47.185	23.593	33.526 ***	0.000
	TDZ	23.185	11.593	16.474 ***	0.000
Differentiation coefficient (Leaf)	6-BA	0.889	0.444	1.091	0.357
	NAA	1.556	0.778	1.909	0.177
	2,4-D	0.222	0.111	0.273	0.764
	TDZ	0.667	0.333	0.818	0.457

Differentiation coefficient (Stem segment with bud) R2 = 0.313 (adjusted R2 = 0.007); Differentiation coefficient (Leaf) R2 = 0.942 (adjusted R2 = 0.916) “***” indicates highly significant difference at the 0.001 level.

**Table 3:** ‘11-C-2’ Root culture results of adventitious buds.

Treatment	Factor A	Factor B	Rooting Rate (%)	Root Number	Root Length (cm)
	Basic Medium	NAA (mg/L)			
B-1	1/4 MS	0.1	95.24 ± 4.12 a	9.87 ± 1.47 c	7.46 ± 0.90 c
B-2		0.2	94.84 ± 4.51 a	7.12 ± 0.40 e	5.87 ± 0.35 e
B-3		0.3	94.44 ± 4.81 a	5.45 ± 0.59 f	4.67 ± 0.11 f
B-4	1/2 MS	0.1	97.62 ± 4.12 a	13.92 ± 0.70 a	9.36 ± 0.71 a
B-5		0.2	97.22 ± 4.81 a	9.71 ± 1.15 c	7.22 ± 0.40 c
B-6		0.3	94.44 ± 4.81 a	9.08 ± 0.43 cd	6.74 ± 0.32 cd
B-7	MS	0.1	97.62 ± 4.12 a	12.18 ± 0.69 b	8.32 ± 0.20 b
B-8		0.2	97.62 ± 4.12 a	8.45 ± 1.07	6.77 ± 0.38 cd
B-9		0.3	94.84 ± 4.51 a	7.86 ± 0.45 de	5.98 ± 0.40 de

The rooting rate, root number, and root length were compared by Duncan method, with results expressed as “mean ± standard deviation”. Differences indicated by different lowercase letters are highly significant (*p* < 0.001), but there was no significant difference among the variation at 5% of One-way ANOVA in rooting rate (*p* = 0.943). Each experimental treatment involved a total of N = 54 plantlets.

**Table 4:** ANOVA of ‘11-C-2’ root culture.

Character	Source of Variance	Sum of Squares	Mean Square	F	*p*
Rooting rate	Basic medium	18.042	9.021	0.547	0.587
	NAA	27.168	13.584	0.823	0.452
Root number	Basic medium	53.295	26.647	39.101 ***	0.000
	NAA	102.348	51.174	75.091 ***	0.000
Root length	Basic medium	14.263	7.132	33.498 ***	0.000
	NAA	31.347	15.674	73.621 ***	0.000

Rooting rate R2 = 0.111 (adjusted R2 = −0.051); Root number R2 = 0.912 (adjusted R2 = 0.896); Root length R2 = 0.907 (adjusted R2 = 0.890); “***” indicates highly significant difference at the 0.001 level.

**Table 5:** Results of visual analyses using plant height as an indicator.

Treatment	Factor A	Factor B	Plant Height (0 d)	Plant Height (5 d)	Plant Height (10 d)	Plant Height (15 d)
	Seedling Refining Time (d)	Substrate for Transplanting				
C-1	1	1:1	3.21 ± 0.10 ab	3.98 ± 0.31 e	4.43 ± 0.23 f	4.97 ± 0.15 e
C-2		1:2	3.26 ± 0.06 ab	4.26 ± 0.32 de	4.97 ± 0.12 cd	5.43 ± 0.15 c
C-3		1:3	3.18 ± 0.12 b	4.11 ± 0.20 e	4.67 ± 0.12 ef	5.17 ± 0.06 cde
C-4	3	1:1	3.12 ± 0.06 b	4.38 ± 0.21 cde	4.72 ± 0.15 de	5.12 ± 0.21 de
C-5		1:2	3.28 ± 0.15 ab	4.78 ± 0.15 bc	5.23 ± 0.06 bc	5.90 ± 0.10 b
C-6		1:3	3.31 ± 0.20 ab	4.59 ± 0.20 bcd	5.07 ± 0.21 c	5.41 ± 0.10 cd
C-7	5	1:1	3.42 ± 0.15 a	4.64 ± 0.35 bcd	5.10 ± 0.10 c	5.37 ± 0.06 cd
C-8		1:2	3.23 ± 0.15 ab	5.27 ± 0.21 a	5.84 ± 0.21 a	6.40 ± 0.10 a
C-9		1:3	3.37 ± 0.12 ab	5.03 ± 0.31 ab	5.47 ± 0.15 b	6.00 ± 0.26 b

The plant heights were compared by Duncan method, with results expressed as “mean ± standard deviation”. Different lowercase letters indicate statistically significant differences (*p* < 0.001). Each experimental treatment involved a total of N = 30 rooted plantlets.

**Table 6:** ANOVA of ‘11-C-2’ seedling refining and transplanting.

Character	Source of Variance	Sum of Squares	Mean Square	F	*p*
Plant height (0 d)	Seedling refining time	0.092	0.046	2.36	0.118
	Substrate for transplanting	0.003	0.001	0.076	0.927
Plant height (5 d)	Seedling refining time	3.387	1.693	28.559 ***	0.000
	Substrate for transplanting	0.896	0.448	7.552 **	0.003
Plant height (10 d)	Seedling refining time	2.749	1.374	58.651 ***	0.000
	Substrate for transplanting	1.562	0.781	33.332 ***	0.000
Plant height (15 d)	Seedling refining time	2.456	1.228	39.137 ***	0.000
	Substrate for transplanting	2.570	1.285	40.943 ***	0.000

Plant height (0 d) R^2^ = 0.181 (adjusted R^2^ = 0.032); Plant height (5 d) R^2^ = 0.767 (adjusted R^2^ = 0.724); Plant height (10 d) R^2^ = 0.893 (adjusted R^2^ = 0.874); Plant height (15 d) R^2^ = 0.879 (adjusted R^2^ = 0.857); “**” indicates highly significant difference at the 0.01 level; “***” indicates highly significant difference at the 0.001 level.

## Data Availability

Data are contained within the article.

## Appendix A

**Table A1:** Range analysis of each dependent variable in the regeneration process.

Dependent Variable	Influencing Factor	k1	k2	k3	R
Differentiation coefficient (Stem segment with bud)	6-BA	6.11	10.44	6.33	4.33
	NAA	7.33	6.56	9.00	2.44
	2,4-D	9.33	7.44	6.11	3.22
	TDZ	8.44	8.11	6.33	2.11
Differentiation coefficient (Leaf)	6-BA	1.44	1.67	1.22	0.44
	NAA	1.78	1.33	1.22	0.56
	2,4-D	1.56	1.44	1.33	0.22
	TDZ	1.56	1.56	1.22	0.33
Rooting rate	Basic medium	94.84	96.43	96.69	1.85
	NAA	96.83	96.56	94.58	2.25
Root number	Basic medium	7.48	10.90	9.50	3.42
	NAA	11.99	8.43	7.46	4.53
Root length	Basic medium	6.00	7.77	7.02	1.77
	NAA	8.38	6.62	5.80	2.58
Plant height (0 d)	Seedling refining time	3.22	3.24	3.34	0.12
	Substrate for transplanting	3.25	3.26	3.29	0.04
Plant height (5 d)	Seedling refining time	4.12	4.58	4.98	0.86
	Substrate for transplanting	4.33	4.77	4.58	0.44
Plant height (10 d)	Seedling refining time	4.69	5.01	5.47	0.78
	Substrate for transplanting	4.75	5.35	5.07	0.60
Plant height (15 d)	Seedling refining time	5.19	5.48	5.92	0.73
	Substrate for transplanting	5.15	5.91	5.53	0.76

k1, k2, and k3 are the average values of different levels of influencing factors of each dependent variable in the regeneration process. The R-value is the range of k1, k2, and k3.

**Table A2:** The interaction effect test of each dependent variable in the regeneration process.

Dependent Variable	The Source	Sum of Squares	Mean Square	F	*p*
Differentiation coefficient (Stem segment with bud)	6-BA × NAA	205.63	25.704	36.526 ***	0.000
Root number	Base medium × NAA	157.076	19.635	27.369 ***	0.000
Root length		46.192	5.774	25.336 ***	0.000
Plant height (5 d)	Seedling refining time × transplanting medium	4.38	0.548	8.167 ***	0.000
Plant height (10 d)		4.373	0.547	21.706 ***	0.000
Plant height (15 d)		5.323	0.665	30.449 ***	0.000

Differentiation coefficient R^2^ = 0.942 (adjusted R^2^ = 0.916); Number of roots R^2^ = 0.924 (adjusted R^2^ = 0.890), root length R^2^ = 0.918 (adjusted R^2^ = 0.882); Plant height (5 d) R^2^ = 0.784 (adjusted R^2^ = 0.688), plant height (10 d) R^2^ = 0.906 (adjusted R^2^ = 0.864), plant height (15 d) R^2^ = 0.931 (adjusted R^2^ = 0.901); *** indicates a significant difference at the 0.001 level.

**Table A3:** Univariate test results of each dependent variable in the regeneration process.

Dependent Variable	Univariate	Level	Sum of Squares	Mean Square	F	*p*
Differentiation coefficient (Stem segment with bud)	6-BA (mg/L)	1	28.222	14.111	20.053 ***	0.000
		1.5	67.556	33.778	48.000 ***	0.000
		2	2.667	1.333	1.895	0.179
	NAA (mg/L)	0.1	6.000	3.000	4.263 *	0.031
		0.2	57.556	28.778	40.895 ***	0.000
		0.3	114.000	57.000	81.000 ***	0.000
Root number	Base medium	1/4 MS	29.609	14.804	20.636 ***	0.000
		1/2 MS	42.007	21.003	29.277 ***	0.000
		MS	32.54	16.27	22.679 ***	0.000
	NAA (mg/L)	0.1	24.949	12.474	17.388 ***	0.000
		0.2	9.887	4.943	6.891 **	0.006
		0.3	20.16	10.08	14.051 ***	0.000
Root length	Base medium	1/4 MS	11.752	5.876	25.784 ***	0.000
		1/2 MS	11.674	5.837	25.613 ***	0.000
		MS	8.502	4.251	18.653 ***	0.000
	NAA (mg/L)	0.1	5.431	2.716	11.916 ***	0.001
		0.2	2.835	1.417	6.22 **	0.009
		0.3	6.579	3.289	14.433 ***	0.000
Plant height (5 d)	Seedling refining time	1 d	0.136	0.068	1.011	0.384
		3 d	0.242	0.121	1.807	0.193
		5 d	0.616	0.308	4.591 *	0.024
	Substrate for transplanting	1:1	0.676	0.338	5.039 *	0.018
		1:2	1.5	0.75	11.188 ***	0.001
		1:3	1.309	0.654	9.762 ***	0.001
Plant height (10 d)	Seedling refining time	1 d	0.429	0.214	8.515 **	0.002
		3 d	0.389	0.194	7.721 **	0.004
		5 d	0.807	0.403	16.015 ***	0.000
	Substrate for transplanting	1:1	0.669	0.334	13.279 ***	0.000
		1:2	1.182	0.591	23.471 ***	0.000
		1:3	0.96	0.48	19.059 ***	0.000
Plant height (15 d)	Seedling refining time	1 d	0.329	0.164	7.525 **	0.004
		3 d	0.909	0.454	20.797 ***	0.000
		5 d	1.629	0.814	37.271 ***	0.000
	Substrate for transplanting	1:1	0.242	0.121	5.542 **	0.013
		1:2	1.402	0.701	32.085 ***	0.000
		1:3	1.109	0.554	25.373 ***	0.000

“*” indicates highly significant difference at the 0.05 level; “**” indicates highly significant difference at the 0.01 level; “***” indicates highly significant difference at the 0.001 level.

**Table A4:** Composition of base medium.

Ingredient	1/4 MS (mg/L)	1/2 MS (mg/L)	MS (mg/L)
KNO_3_	475	950	1900
NH_4_NO_3_	412.5	825	1650
KH_2_PO_4_	42.5	85	170
MgSO_4_·7H_2_O	92.5	185	370
CaCl_2_·2H_2_O	110	220	440
KI	0.83		
H_3_BO_3_	6.2		
MnSO_4_·H_2_O	22.30		
ZnSO_4_·7H_2_O	8.6		
Na_2_MoO_4_·2H_2_O	0.25		
CuSO_4_·5H_2_O	0.025		
CoCl_2_·6H_2_O	0.025		
Na_2–_EDTA	37.3		
FeSO_4_·4H_2_O	27.8		
C_6_H_12_O_6_	100		
C_2_H_5_NO_2_	2		
C_12_H_17_ClN_4_OS·HCl	0.1		
C_8_H_12_ClNO_3_	0.5		
C_6_H_5_NO_2_	0.5		
Sucrose	30,000		
Agar	7000		

The pH range of different base mediums was 5.6~5.8. To prepare the media, weigh 38.4 g of 1/4 MS powder, 39.45 g of 1/2 MS powder, and 41.74 g of MS powder. Dissolve each in 1000 mL of distilled water, adjust the pH to 5.7 using 1 M NaOH or HCl, and then autoclave at 116 °C for 30 min. The basic media used were products from Qingdao Haibo Biotechnology Co., China, with product codes HB8469-8, HB8469-6, and HB8469.

**Table A5:** Orthogonal experimental design for apical meristem differentiation.

Treatment	6-BA (mg/L)	NAA (mg/L)	2,4-D (mg/L)	TDZ (mg/L)
A-1	1	0.1	0	0
A-2	1	0.3	0.3	0.1
A-3	1	0.5	0.1	0.3
A-4	1.5	0.1	0.3	0.3
A-5	1.5	0.3	0.1	0
A-6	1.5	0.5	0	0.1
A-7	2	0.1	0.1	0.1
A-8	2	0.3	0	0.3
A-9	2	0.5	0.3	0

All basic media were MS.

**Table A6:** Orthogonal experimental design for rooting culture.

Treatment	Base Medium	NAA (mg/L)
B-1	1/4 MS	0.1
B-2		0.2
B-3		0.3
B-4	1/2 MS	0.1
B-5		0.2
B-6		0.3
B-7	MS	0.1
B-8		0.2
B-9		0.3

The component of base medium is shown in Table A4.

**Table A7:** Orthogonal experimental design for acclimatization and transplantation.

Treatment	Seedling Refining Time (d)	Substrate for Transplanting
C-1	1	1:1
C-2		1:2
C-3		1:3
C-4	3	1:1
C-5		1:2
C-6		1:3
C-7	5	1:1
C-8		1:2
C-9		1:3

The peat used is a product from PINDSTRUO, and the vermiculite is white vermiculite from Xinjiang with a particle size of 1–3 mm. The components of the transplanting soil are measured by weight using a balance, with the soil mixture consisting of peat and vermiculite in a specified ratio. And, they do not need high-temperature sterilization treatment, and can be directly used to simulate the field growing environment.
